# The treatment of non-malarial febrile illness in Papua New Guinea: findings from cross sectional and longitudinal studies of health worker practice

**DOI:** 10.1186/s12913-016-1965-6

**Published:** 2017-01-05

**Authors:** Olga P. M. Saweri, Manuel W. Hetzel, Ivo Mueller, Peter M. Siba, Justin Pulford

**Affiliations:** 1 Papua New Guinea Institute of Medical Research (PNGIMR), PO Box 60, Goroka, EHP 441 Papua New Guinea; 2 Swiss Tropical and Public Health Institute, PO Box, 4002, Basel, Switzerland; 3 University of Basel, Petersplatz 1, 4003, Basel, Switzerland; 4 Barcelona Centre for International Health Research (CRESIB, Hospital Clínic-Universitat de Barcelona), Barcelona, Spain; 5 Walter and Eliza Hall Institute of Medical Research, Melbourne, Australia; 6 Liverpool School of Tropical Medicine, Liverpool, UK

**Keywords:** Malaria, Febrile, Treatment, Diagnosis, Prescription, Health worker practice

## Abstract

**Background:**

The Papua New Guinea Department of Health recently shifted from a presumptive to a ‘test and treat’ malaria case management policy. This shift was supported by the widespread introduction of malaria rapid diagnostic tests in health facilities across the country. Health workers received training and job-aids detailing how to conduct and interpret a malaria rapid diagnostic test and how to treat test positive cases; however, little instruction on treating non-malaria febrile cases was provided. Accordingly, this study examined health worker case management of non-malarial febrile patients in the 12-month period immediately following the introduction of the revised malaria case management policy.

**Methods:**

Data were collected from a country-wide cross-sectional survey of febrile case management at randomly selected health facilities and from longitudinal surveillance at sentinel health facilities. Analysis was restricted to febrile patients who tested negative for malaria infection by rapid diagnostic test (N=303 and 5705 outpatients, respectively).

**Results and Discussion:**

96.8% of non-malarial febrile patients received a diagnosis in the longitudinal sample, compared to 52.4% of the cross-sectional sample. Respiratory tract infections were the most commonly reported diagnoses. Over 90% of patients in both samples were prescribed one or more medications, most commonly an analgesic (71.3 & 72.9% of the longitudinal and cross-sectional samples, respectively), some form of antibiotic (72.7 & 73.4%, respectively) and/or an anthelminthic (17.9 & 16.5%, respectively). Prescribing behaviour was adherent with the recommendations in the standard treatment guidelines in fewer than 20% of cases (longitudinal sample only).

**Conclusion:**

Many non-malarial febrile patients are not provided with a diagnosis. When diagnoses are provided they are typically some form of respiratory tract infection. Antibiotics and analgesics are widely prescribed, although medications prescribed rarely adhere to the Papua New Guinea standard treatment guidelines. These findings indicate that Papua New Guinea health workers require support for non-malarial febrile illness case management.

**Electronic supplementary material:**

The online version of this article (doi:10.1186/s12913-016-1965-6) contains supplementary material, which is available to authorized users.

## Background

The last 5 to 10 years has seen a substantial increase in the availability and use of malaria Rapid Diagnostic Tests (mRDTs) in many parts of the world [1]. Consistent with these global trends, Papua New Guinea (PNG) with support from a Round 8 Global Fund grant has procured over three million mRDTs since 2010 [2]. This supports a revised national malaria treatment policy (NMTP) stipulating that all fever or suspected malaria cases be tested for malaria infection by microscopy or mRDT [3]. Internationally, the scale-up of mRDT availability has often led to substantial reductions in antimalarial prescription as health workers shift from a presumptive to a ‘test and treat’ malaria case management approach [4–6]. There is some evidence to suggest a similar change in health worker practice is occurring in PNG. A recent study found that antimalarial prescriptions to febrile patients declined from 96.4% of cases before the introduction of the test and treat policy to 39.0% in the 12-month period immediately post-implementation [7]. The availability of mRDTs increased from 8.9% of surveyed facilities to 53.4% across the same time period.

Whilst mRDTs can confirm or rule out malaria infection, they provide no support to health workers for diagnosing and treating non-malarial fevers. Fever is an unspecific syndrome and diagnostic tools to assist in the accurate identification of its aetiology in mRDT negative patients are scarce in resource-poor settings [8]. The historic practice of presumptive malaria diagnosis may further undermine the quality of non-malarial febrile case management as the health workforce has little acquired experience in the clinical diagnosis and treatment of non-malarial febrile patients [9]. Many febrile patients attending PNG health facilities test negative for malaria infection and the malaria burden is declining in the general population [10, 11]. Thus, the health workforce is increasingly, and quite suddenly, required to respond to febrile illnesses shown to be non-malarial in origin via mRDT.

A health worker training program was carried out in PNG pre-implementation of the new NMTP. This program provided detailed instruction on mRDT use and strongly emphasised the importance of restricting antimalarial prescription to test-confirmed cases [12]. Nevertheless, the training program provided relatively little detail on non-malarial febrile case management beyond reference to an Integrated Management of Childhood Illness (IMCI) flowchart. No instruction on the management of non-malarial febrile illness in adolescent or adult patients was provided. The PNG National Department of Health and the Paediatric Society of PNG issues standard treatment guidelines which outline recommended management of a range of common illnesses, including non-malarial febrile illness [13, 14]. Apart from these reference manuals, no other training, resources or support have been specifically provided to assist the PNG healthcare workforce manage the increasing number of non-malarial febrile illness cases identified in the context of the new NMTP. Accurate diagnosis of non-malarial febrile illness is further comprised by the absence of on-site laboratory support at most primary health care facilities in PNG [15]. The general lack of curative treatments for most viral febrile illnesses may be an additional obstacle to implementing accurate evidence-based treatment.

This paper examines how the PNG primary healthcare workforce diagnosed and treated non-malarial febrile patients in the period immediately following the widespread introduction of mRDTs. The primary research questions included: what percentage of non-malarial febrile patients are provided a recorded diagnosis? What diagnoses are being recorded? What medications are being prescribed? And to what extent do the medications prescribed adhere with national standard treatment guidelines?

## Methods

This paper presents selected data from a multifacted evaluation of the PNG National Malaria Control Program (NMCP), 2009–2014. A full description of the NMCP evaluation is available elsewhere [16]. Reported data were obtained from longitudinal, outpatient surveillance in seven sentinel health facilities and a cross sectional survey completed in 36 randomly selected health facilities across the country. The longitudinal dataset provided a large number of febrile cases, although the respective case management of these patients was provided by a relatively small number of health workers working within a quasi-research environment. The cross sectional dataset provided fewer clinical cases, but these cases were clinically managed by a greater number of health workers from across a greater number of health facilities in a routine clinical environment. The datasets are considered complementary as, together, they provide clinical case management records pertaining to non-malaria febrile illness for over 6000 patients from 43 health facilities.

### Outpatient surveillance

#### Study sites

Outpatient surveillance was variously initiated in seven sentinel primary health care facilities across PNG between January 2010 and July 2011. The primary aim of the surveillance was to assess trends in malaria morbidity. The seven facilities were purposively selected from a sampling frame of operational public-sector health centres provided by the PNG Department of Health according to their accessibility, their functionality, their geographical spread and the local malaria epidemiology and were located in Balimo (Western province), East Cape (Milne Bay province), Karimui (Chimbu province), Sausi (Madang province), Dreikikir (East Sepik province), Lemakot (New Ireland province) and Arawa (Autonomous Region of Bougainville). For the purpose of this paper, focus is placed on data collected in the calendar year 2012 as the revised NMTP was implemented in November, 2011.

#### Procedure

Daily surveillance was carried out at each health facility during regular opening hours (Monday to Friday, 7.45 am-4.06 pm). Outpatients presenting with fever or a history of fever, in the past 3 days, were referred to a study nurse who then completed an mRDT, prepared a thick and thin blood smear on a microscopy slide and measured haemoglobin (Hb) level using a Hemocue Hb 201+ analyser (HemoCue AB, Ängelholm, Sweden). Demographic details of the patient were recorded in a one-page case report form alongside clinical signs and symptoms, previous health facility attendance and drug intake, axillary temperature, weight and results of the mRDT and Hb measurement. The patient was then transferred to a member of the respective facility’s health work force for further examination, diagnosis and treatment following routine procedures. The resulting diagnosis and any prescription by the health worker were recorded on the case report form. Oral informed consent was obtained from all patients prior to participation.

### Cross sectional survey

#### Study sites

The sample consisted of primary health care facilities randomly selected as part of a countrywide cross sectional health facility survey in which at least one instance of febrile case management has been observed by a member of the research team. The sampling frame consisted of all primary health care facilities operational at the time of the survey. Health facilities were stratified by province and selected using a simple random sampling procedure. Sample size was determined by logistical considerations.

#### Procedure

The survey was carried out between June and November 2012. Research officers spent between 1 to 5 days at each participating health facility (depending on size). Up to four different survey instruments were completed at each health facility, although this paper only reports data obtained from the non-participant observation of febrile case management (described below). Oral informed consent was sought from the officer in charge at all participating health facilities and from all participating clinicians and patients prior to clinical observation. More details of the general survey methodology have been published elsewhere [7].

#### Survey instrument

A structured checklist was designed to record observed features of the clinical case management of patients presenting with fever or a recent history of fever. The instrument was divided into discrete sections including consultation and diagnosis, prescription and treatment counseling. The content of each section was informed with input from experienced medical- and medical research- professionals. The instrument was completed by a trained research officer who would passively observe the management of fever patients from the point of initial contact with a health professional until service exit or admission onto a treatment ward. During the course of this observation, the research officer recorded on the structured clinical observation instrument whether specified actions did or did not occur and recorded the content of specific actions (e.g., whether an mRDT was conducted and, if yes, the outcome). Eligible patients were identified upon first contact with a health worker or, if circumstances allowed, by screening in the waiting area prior to first contact with a health worker.

### Data analysis

All data were double entered into DMSys version 5.1 (Sigma Soft International). Stata versions 11/IC and 12/SE were used for descriptive data analysis and to assess health worker adherence to prescription guidelines. The analysis of health worker adherence to prescription guidelines was limited to the ten most frequently recorded diagnoses and to patients with a single recorded diagnosis. Patients with multiple diagnoses were not included as it was not possible to determine which medications were prescribed for which diagnosis. To be rated ‘adherent’ the medications prescribed were required to be consistent with those listed against the specified diagnosis in either the Papua New Guinea Standard Treatment Guidelines for Adults [14] or Children [13], as listed in Table 1. A rating of ‘partially adherent’ was given when at least one (but not all) of the listed medications for the specified diagnosis were prescribed or the same class of medication was prescribed, but not the recommended drug. A rating of ‘non-adherent’ was given when the medication/s prescribed was/were neither the recommended drug nor the same class of medication as stated in the aforementioned manuals. The analysis of health worker adherence to prescription guidelines did not account for drug dosages and was not applied to the CS sample due to the large number of cases without a recorded diagnosis. Between-group differences in the mean number of medications prescribed and type of prescription were examined by two-tailed *t*-tests and chi-square, respectively, in the CS sample.

**Table 1:** Recommended medications for specified diagnoses according to the Papua New Guinea Standard Treatment Guidelines for Adults and Children

Diagnosis	Recommended medications	
	Adults	Children
Anaemia^a^	Fefol; Albendazole; if spleen is very large, Chloroquine	Antimalarial + Albendazole + Folic Acid + Iron (for severe cases a blood transfusion)
Cough^b^	Amoxicillin + Paracetamol (if simple cough)	Amoxicillin
Diarrhoea^c^	Treat dehydration with Oral Rehydration Salts (ORS); in severe cases treat intravenously with ORS	Treat dehydration with Oral Rehydration Salts (ORS); in severe cases treat intravenously with ORS
Gastroenteritis^c^	Treat dehydration with Oral Rehydration Salts (ORS); in severe cases treat intravenously with ORS	Treat dehydration with Oral Rehydration Salts (ORS); in severe cases treat intravenously with ORS
Influenza-like-illness	Amoxicillin (bacterial infection) + Paracetamol (fever)	Amoxicillin (bacterial infection) + Paracetamol (fever)
Malaria^d^	*P.falciparum*: Artemether Lumefantrine (AL); *P.vivax*: AL + Primaquine	*P.falciparum*: Artemether Lumefantrine (AL); *P.vivax*: AL + Primaquine
Otitis Media^e^	Amoxicillin + Paracetamol (acute otitis media); Co-trimoxazole (for pus in chronic Otitis media)	Amoxicillin + Paracetamol (acute otitis media); Co-trimoxizole (if pus/discharge after 1 week of treatment); Boric Acid ear drops (Chronic Otitis Media)
Pneumonia^f^	Paracetamol (chest pains) + Amoxicillin (mild, moderate); Benzyl-penicillin (severe); Chloramphenicol (severe); gentamicin (severe); ceftriaxone (severe) + Oxygen (severe) + nebulised salbutamol (for wheezing); salbutamol inhaler (for wheezing)	Chloramphenicol (severe pneumonia) + Oxygen (if child is cyanosed/grunting/in heart failure/restless/drowsy/stopping breathing); Benzyl Penicillin (moderate pneumonia); Amoxicillin (mild pneumonia)
URTI^g^	Amoxicillin (bacterial infection) + Paracetamol (fever)	Amoxicillin (bacterial infection) + Paracetamol (fever)
Worms	Albendazole	Albendazole

^a^Includes severe anaemia, anaemia acquired in pregnancy, anaemia acquired after birth and non-specific anaemia

^b^Includes general cough, simple cough and productive cough

^c^A rating could not be made in the majority of cases due to insufficient diagnostic information (e.g., diarrhoea would be treated in different ways depending on the cause, but the cause was rarely stated)

^d^Includes uncomplicated malaria, severe malaria and non-specific malaria

^e^Includes both acute and chronic otitis media as well as non-specific otitis media

^f^Includes moderate, mild and severe pneumonia

^g^Upper Respiratory Tract Infection

## Results

### Outpatient surveillance

#### Sample

A total of 5705 patients with fever or recent experience of fever tested negative for malaria by mRDT across the seven sentinel sites in 2012. Of these patients, 50.9% were female, 42.3% were <5 years of age, 18.7% were between 5 and 15 years of age and 39.0% were 16 years of age or older.

#### Diagnosis

A diagnosis was recorded for 96.8% (5521/5705) of the outpatient surveillance (OS) sample. Of outpatients with a recorded diagnosis, 88.9% (4906/5521) had one recorded diagnosis and 11.1% (615/5521) had two or more. Overall, a total of 6152 diagnoses were recorded of which 87.6% (5391/6152) appear in the Papua New Guinea Standard Treatment Guidelines for Adults [14] or Children [13]. A full list of the recorded diagnoses is available in Additional file 1. The ten most frequently recorded diagnoses, accounting for 72.6% (4469/6152) of all diagnoses, included: Pneumonia (*n* = 824), Cough (*n* = 790), Respiratory Tract Infection (*n* = 613), Malaria (*n* = 510), Anaemia (*n* = 452), Fever of Unknown Origin (*n* = 399), Otitis Media (*n* = 286), Diarrhoea (*n* = 274), Flu (*n* = 233) and Worms (*n* = 88).

All participants included in the OS sample were tested for malaria and anaemia using diagnostic tests. These were the only two diagnostic test results routinely recorded that allow the accuracy of the respective diagnosis to be assessed. By definition, all participants included in the OS sample tested negative for malaria infection by mRDT; however, 8.9% (510/5705) were given a recorded diagnosis of malaria all of which would be considered incorrect according to mRDT result assuming absence of signs of severe malarial disease (7/510 malaria diagnose were recorded as ‘severe malaria’). Anaemia was defined, by the Papua New Guinea Standard Treatment Guidelines, as any patient presenting with Hb less than 10 g/dL. A Hb level lower than 5 g/dL indicates severe anaemia. Overall, 27.4% (1561/5705) of the OS sample tested positive for either moderate (*n* = 1481) or severe anaemia (*n* = 80). Of these 1561 patients, 24.9% were formally diagnosed with anaemia.

#### Medication

Ninety-six point five percent (5508/5705) of the OS sample were prescribed one or more medications, the number, percentage and type of which are depicted in Table 2. The mean number of medications prescribed (analysis restricted to patients prescribed at least one medicine) was 1.8 (Median 1; IQR 1–2). Overall, antibiotics were the most commonly prescribed medication (72.7%), followed by analgesics (71.3%) and anthelminthics (17.9%). One or more antimalarial medications were prescribed to 13.2% (753/5705) of the OS sample, the most common of which were sulphadoxine/pyrimethamine (SP) (8.1%), chloroquine (4.2%) and amodiaquine (3.6%). 58.6% (441/753) of these patients received some form of antimalarial combination, the most common of which were SP plus chloroquine (21.1%), and SP plus amodiaquine (19.1%). Artemether monotherapies were prescribed in 2.9% (22/753) of cases.

**Table 2:** Number, percent and type of prescribed medications by survey type

Drug Classification & Type	Outpatient Surveillance (*N* = 5705)		Cross Sectional (*N* = 303)	
	n	%	n	%
Antimalarial	753	13.2	57	18.8
Amodiaquine	203	3.6	21	6.9
Artemether	160	2.8	7	2.3
Artemether-Lumefantrine	52	0.9	3	1.0
Artesunate	12	0.2	1	0.3
Chloroquine	240	4.2	20	6.6
Dihydroartemisinin-Piperaquine	1	0.02	0	0
Doxycycline	91	1.6	2	0.6
Primaquine	13	0.2	1	0.3
Quinine	6	0.1	0	0
Sulfadoxine-Pyrimethamine	461	8.1	42	13.9
Antibiotic	4147	72.7	223	73.4
Amoxicillin	1429	25.0	124	40.9
Co-trimoxazole	1179	20.7	70	23.1
Benzylpenicillin	478	8.4	42	13.9
Chloramphenicol	129	2.3	12	4.0
Metronidazole	32	0.6	3	1.0
Erythromycin	49	0.9	1	0.3
Eye ointment	20	0.4	1	0.3
Analgesic/antipyretic	4068	71.3	221	72.9
Aspirin	261	4.6	28	9.2
Paracetamol	3765	66.0	190	62.7
Indomethacin	32	0.6	3	1.0
Haematinics	438	7.7	10	3.3
Ferrous salt + folic acid	419	7.3	9	2.9
Interferon	19	0.3	1	0.3
Gastro Intestinal	19	0.3	5	1.7
Cimetidine	4	0.1	1	0.3
Aluminium-Hydroxide	15	0.3	3	1.0
Anti-diabetic	3	0.1	0	0
Metaformin	3	0.1	0	0
Anticonvulsant	2	0.04	1	0.3
Phenobarbitone	0	0	1	0.3
Antiprotozoal	343	6.0	17	5.6
Tinidazole	343	6.0	16	5.3
Anthelminthic	1022	17.9	50	16.5
Albendazole	1022	17.9	42	13.9
Corticosteroid	0	0	1	0.3
Prednisolone	0	0	1	0.3
Bronchodilator	76	1.3	0	0
Salbutamol	74	1.3	0	0
Antifungal	12	0.2	1	0.3
Dermatological lotion	7	0.1	1	0.3
Dermatological oral drugs	5	0.1	0	0
Cardio-vascular	3	0.1	0	0
Methyldopa	3	0.1	0	0
Rehydration	82	1.4	0	0
Oral rehydration salts	73	1.3	1	0.3
Glucose	9	0.2	0	0

#### Health worker adherence to prescription guidelines

As shown in Table 3, a total of 3249 patients met the inclusion criteria for this analysis of whom 16.6% received a prescription in adherence with the standard treatment guidelines for their respective diagnosis. The majority of prescriptions (56.3%) were rated ‘partially adherent’, 21.8% ‘non-adherent’ and a rating was unable to be made in 5.4% of cases.

**Table 3:** Health worker adherence to prescription guidelines relative to recorded diagnosis

Diagnosis	Number		Adherence Rating (%)		
		Adherent	Partially Adherent	Non-adherent	Unable to Rate
Anaemia^a^	270	3.7	90.7	5.6	0
Cough^b^	639	8.5	37.4	54.1	0
Diarrhoea^c^	174	0	36.8	0	63.2
Gastroenteritis^c^	71	0	19.7	0	80.3
Influenza-like-illness	207	2.9	36.7	57	3.4
Malaria^d^	346	5.2	83.2	11.6	0
Otitis Media^e^	238	30.3	68.9	0.8	0
Pneumonia^f^	715	38.7	59.6	1.7	0
URTI^g^	526	15.8	52.1	32.1	0
Worms	63	31.7	60.3	7.9	0
Total	3249	16.6	56.3	21.8	5.4

^a^Includes severe anaemia, anaemia acquired in pregnancy, anaemia acquired after birth and non-specific anaemia

^b^Includes general cough, simple cough and productive cough

^c^A rating could not be made in the majority of cases due to insufficient diagnostic information (e.g., diarrhoea would be treated in different ways depending on the cause, but the cause was rarely stated)

^d^Includes uncomplicated malaria, severe malaria and non-specific malaria

^e^Includes both acute and chronic otitis media as well as non-specific otitis media

^f^Includes moderate, mild and severe pneumonia

^g^Upper Respiratory Tract Infection

Table 4, drawing on the same sample as presented in Table 3 and further limited to those patients for whom it was possible to provide an ‘adherence’ rating (*n* = 3075), presents the percentage of participants for whom: according to the respective prescription protocols in the PNG standard treatment guidelines, (1) should have been prescribed an antibiotic and were not (under prescription); and (2) should not have been provided an antibiotic and were (over prescription). As shown, 18.7% of patients in the five diagnostic categories in which an antibiotic should have been prescribed did not receive an antibiotic. Conversely, 41.0% of patients in the five diagnostic categories in which an antibiotic should not have been prescribed did receive an antibiotic.

**Table 4:** Antibiotic prescription relative to diagnosis, according to Papua New Guinea Standard Treatment Guidelines

Diagnosis	Number	Antibiotic Prescription (%)		
		Required	Under^a^	Over^b^
Anaemia	270	NO	-	54.8
Cough	639	YES	37.4	-
Diarrhoea^C^	64	NO	-	92.2
Gastroenteritis^C^	14	YES	0	-
Influenza-like-illness^C^	200	NO	-	37.0
Malaria	346	NO	-	19.1
Otitis Media	238	YES	2.9	-
Pneumonia	715	YES	3.1	-
URTI	526	YES	24.9	-
Worms	63	NO	-	63.5
Total	3075	-	18.7	41.0

^a^Percentages only calculated for patients in those diagnostic categories in which the PNG standard treatment quidelines recommend an antibiotic prescription (*n* = 2132)

^b^Percentages only calculated for patients in those diagnostic categories in which the PNG standard treatment quidelines do not recommend an antibiotic prescription (*n* = 943)

^c^Analysis limited to those participants for whom an ‘adherence rating’ (Table 2) could be made

### Cross sectional survey

#### Sample

A total of 303 febrile patients tested negative for malaria by mRDT or microscopy. Of these patients, 50.8% were female, 47.9% were <5 years of age, 19.5% were between 5 and 15 years of age and 32.7% were 16 years of age or older. These patients were collectively observed across 36 health facilities in 17 provinces.

#### Diagnosis

A diagnosis was only sought for mRDT negative patients not prescribed an antimalarial (*n* = 246) as those prescribed antimalarials (*n* = 57) were assumed to have been diagnosed with malaria despite the mRDT result. Of these 246 patients, a diagnosis was recorded in 52.4% of cases. The specified diagnoses indicated some form of respiratory tract infection (*n* = 80), general body aches or headache (*n* = 28), fever (*n* = 11), diarrhoea (*n* = 9), ear infection (*n* = 7), shortness of breath (*n* = 7), sore mouth/throat (*n* = 5), influenza (*n* = 4), viral infection (*n* = 3), malaise (*n* = 3), vomiting (*n* = 2), food poisoning (*n* = 2) and one each of measles, tuberculosis, anaemia, gastroenteritis, swelling, bleeding eye, pregnancy and hypertension.

Of the 117 mRDT negative patients without a recorded diagnosis (or prescribed antimalarials), 94.0% (110/117) were prescribed some form of medication suggesting the health worker formed a clinical opinion as to the cause of the febrile illness even if this opinion was not formally recorded or reported to the patient.

#### Medication

97.0% (294/303) of the CS sample were prescribed one or more medications, the number, percentage and type of which are listed in Table 2. The mean number of medications prescribed (analysis restricted to patients prescribed at least one medicine) was 2.1 (Median 2; IQR 2–3). Overall, antibiotics were the most commonly prescribed medication (73.4%) followed by analgesics (72.9%). One or more antimalarial medications were prescribed to 18.8% (57/303) of the CS sample despite the negative mRDT result. The most commonly prescribed antimalarials were SP (13.9%), amodiaquine (6.9%) and chloroquine (6.6%). The recommended first line medication for uncomplicated malaria, artemether-lumefantrine, was prescribed in 1.0% (3/303) of cases. 13.9% (42/303) of these patients received some form of antimalarial combination, the most common of which were chloroquine plus SP (6.6%), amodiaquine plus SP (5.0%) and artemether/artesunate plus SP (1.7%). Artemether monotherapies were prescribed in less than 1.0% (2/303) of cases.

Excluding patients prescribed an antimalarial (for whom diagnostic information was not sought), there was no statistically significant difference in the mean number of medications prescribed to mRDT negative patients with a recorded diagnosis as compared to those without a recorded diagnosis (2.0 vs. 1.9; *t* = −0.3, *p* = 0.76). Furthermore, mRDT negative patients not provided a diagnosis were as likely to receive an antibiotic (75.9% vs. 73.1%; *χ* ^2^ = 0.7, *p* = 0.42) or analgesic (71.6% vs. 76.2%; *χ* ^2^ = 0.7, *p* = 0.41) as those with a recorded diagnosis suggesting prescribing behaviour was similar irrespective of whether a formal diagnosis was recorded or not.

## Discussion

This paper presents data pertaining to the clinical case management of non-malarial febrile patients in the 12-month period immediately following the discontinuation of a treatment protocol in which most febrile patients were presumptively treated with antimalarials. Data were derived from longitudinal surveillance in seven outpatient sentinel health facilities and from a cross sectional survey in 36 health centres across 17 provinces in PNG.

Findings from the OS sample indicate that the vast majority of non-malarial febrile patients had a formal diagnosis recorded, in the CS sample this was little more than 50%. As the latter comprised a greater number of health workers, and as these health workers were not prompted to provide a diagnosis (as was the case in the OS sample), then the CS percentage may be the more accurate reflection of standard practice. Respiratory tract infections, often pneumonia, were the most common diagnosis provided across both datasets. Diagnoses of diarrhoea and general body aches were also prominent across samples, although at a substantially lower frequency. Respiratory tract infections and diarrhoea have historically been reported at a high frequency on the PNG National Health Information System [17] and are ranked highly in PNG burden of disease estimates [18]. Thus, the specified diagnoses of non-malarial febrile illness are largely consistent with existing data sources.

It was not possible to determine whether the recorded diagnoses were accurate or not in the context of this study, with the exception of malaria and anaemia. However, the small number of studies that have examined the aetiology of non-malarial febrile illness in PNG have identified dengue as a cause in approximately 10% of cases [19, 20] and a chikungunya virus outbreak was detected in PNG at the time the reported data were collected [21, 22]. A recent review of non-malarial febrile illness in the neighbouring South East Asia region further identified dengue, typhus and *Leptospira* spp. as the most commonly reported pathogens across 146 studies meeting the inclusion criteria [23]. As neither dengue, chikungunya, typhus or leptospira spp. were diagnosed in a single case in either sample, as malaria was diagnosed in between 8.9 to 18.8% of mRDT-negative cases and as anaemia was substantially underdiagnosed despite the availability of a Hb-measurement in the OS sample, there is reason to question the accuracy of recorded diagnoses. The limited diagnostic/laboratory support available in primary health care facilities in PNG restricts the degree to which health workers can investigate the causes of febrile illnesses and may consequently influence the accuracy of diagnoses provided. The threat of misdiagnosis may, therefore, be considered a result of limitations in the broader healthcare system rather than health worker ability per se.

Prescription patterns were consistent across datasets. Over 90% of patients in both the OS and CS sample were prescribed one or more medications, most commonly some form of antibiotic, analgesic and/or anthelminthic. Antimalarials were provided in 18.8 and 13.2% of OS and CS cases, respectively, despite the negative mRDT result. This rate of antimalarial prescription to mRDT negative patients is lower than that reported in other settings [24–26] and represents a substantial reduction in the rate of antimalarial prescription to febrile patients compared to the practice observed prior to the change in treatment protocol [27]. Thus, while not fully compliant with the revised protocol, PNG health workers have seemingly made radical and appropriate adjustments to their antimalarial prescription practices in a relatively brief time frame and perhaps at a faster rate than their international peers in similar circumstances.

The adherence analysis indicated that the prescription provided was consistent with that recommended in the respective PNG standard treatment manual for the specified diagnosis in fewer than 20% of cases (OS sample only). The majority of prescriptions (56.3%) were rated ‘partially adherent’, indicating that at least one, but not all, of the recommended medications was provided or the correct class of medication was provided, but not the recommended drug. It was not possible to determine what influence the availability of the respective medications played in this outcome. This result, therefore, most likely reflects limitations in both medication availability and health worker practice, yet it remains the case that the medications provided were rarely in full accordance with those recommended for the diagnoses given. A previous study, not restricted to mRDT negative cases, reported ‘appropriate’ prescription rates (based on diagnosis) of between 62.1–69.6% in two secondary care settings and an outpatient facility in PNG [28]. Thus, the relatively poor adherence to prescription guidelines reported in this study suggests that PNG health workers may be less likely to provide a recommended prescription when treating non-malarial febrile illness patients. However, in the absence of an expert reference diagnosis in the context of this study, the extent and consequences of any potential misdiagnosis and non-recommended prescription cannot be judged.

Previous studies have reported similar rates of antibiotic prescription to mRDT negative patients as reported here, from 61.4% in Uganda [29] to 78% in Tanzania [30]. Accordingly, the rates may be considered somewhat normative in a low income country setting in which a shift from presumptive to parasitological confirmed malaria diagnosis has recently taken place. Nevertheless, the rate of antibiotic prescription is high and raises the possibility that primary health care workers in PNG may be substituting antimalarial medication with antibiotic medication in response to the revised NMTP, as has been previously suggested [6, 9]. The potential over-prescription of antibiotics cannot be reliably established in the absence of an expert reference diagnosis including, in some instances, additional diagnostic testing; nevertheless, a recent study reported unnecessary antibiotic prescription in 29% of 6969 observed illness episodes in outpatient health services in PNG [31] which is somewhat consistent with the rate of antibiotic over prescription (41%) reported herein. Furthermore, an analysis of medication prescription to malaria mRDT *positive* patients in the CS survey [7] found that out of 54 mRDT positive patients, 98.2% were prescribed an antimalarial, yet only 14.8% were prescribed an antibiotic. This would indicate that health workers are selectively (and often unnecessarily) prescribing antibiotics to mRDT negative cases as opposed to routinely providing them to all febrile patients.

This study was not without limitation. Firstly, the fact that (to a large extent) the accuracy of the reported diagnoses was not able to be assessed in either the OS or CS sample remains an important knowledge gap. Whilst assessing the accuracy of non-malarial febrile illness was not an aim of the study, without this essential information it is difficult to reliably assess the quality of health worker practice. A further limitation is that the study did not take into consideration medication supplies at the respective health facilities in either survey. The supply of anti-malarial drugs or other medications could have an implied effect on the prescribing behaviour of health workers [32]. In addition, health workers in the OS sample were required to complete a diagnosis and medication section on a research-specific case report form which is not reflective of normal practice and health workers in the CS sample were actively observed during their respective febrile case consultations by a member of a research team. This, too, may have influenced health worker practice. The health workers in both surveys were predominantly nurses and community health workers. Therefore, the conclusions drawn concerning health worker behaviour are not a complete depiction of all health worker cadres in PNG.

The respective datasets also had their inherent weaknesses. The OS analyses were based on a large number of patient cases, yet they had been collectively treated by a relatively small number of health workers. Conversely, the CS analyses were based on a smaller number of patients, yet they had been treated by a larger number of health workers from a more diverse array of health facilities. Triangulating findings from the two datasets, as was done here, overcomes their respective limitations in part. Despite these limitations, the collective sample size of both patients and health workers, the relatively robust sampling and study protocols, the geographical spread of participating health centres and the general agreement between the two datasets with respect to reported diagnosis and prescription practice suggests the reported findings could reasonably be generalised to health centres across PNG.

## Conclusions

The findings indicate health workers predominantly adhere to mRDT negative results, determine alternative diagnoses in most cases and provide medications other than antimalarials. However, the findings further suggest that a large proportion of non-malarial febrile patients are not being provided a diagnosis, the diagnoses that are given may not necessarily be accurate, the medications prescribed rarely fully adhere to those recommended for the specified diagnosis and that antibiotics are likely to be overprescribed. All of these factors strongly indicate a need for intensive and continuing health worker support in the diagnosis and management of non-malarial fevers and for thorough scientific investigation and reporting on the aetiology of non-malarial fevers across the various regions of PNG.

Interventions based on training and/or the provision of rapid diagnostic tests have demonstrably improved non-malarial febrile case management [33], and interventions proven successful in improving malaria case management, such as text message reminders [34], would seemingly be easily transferable. General health systems strengthening in low income country contexts such as PNG is equally essential to sustained improvement in health worker performance [35]. As multiple support and supervisory mechanism are typically required to improve health worker practice [36], strengthening non-malarial febrile case management in primary health care contexts in PNG will likely require a package of health worker and health system strengthening interventions, variously focusing on: further reducing antimalarial prescription to malaria mRDT negative patients; encouraging formal diagnosis and the use of clinical and available laboratory resources to inform diagnosis; rational use of antibiotics; and adherence to recommended treatment guidelines when a diagnosis is made.

## Additional file

Additional file 1: Full list of recorded diagnoses for the outpatient surveillance sample. (DOCX 21 kb)

## Abbreviations

CS: Cross-sectional
Hb: Haemoglobin
IMCI: Integrated management of childhood illness
IQR: Interquartile range
mRDT: malaria rapid diagnostic test
NMCP: National Malaria Control Program
NMTP: National Malaria Treatment Protocol
OS: Outpatient longitudinal surveillance
PNG: Papua New Guinea
SP: Sulfadoxine-pyrimethamine
