# Supplementary material for: The treatment of non-malarial febrile illness in Papua New Guinea: findings from cross sectional and longitudinal studies of health worker practice
Source: BMC Health Serv Res. 2017 Jan 5;17:10. doi: 10.1186/s12913-016-1965-6 (PMC5217620; doi:10.1186/s12913-016-1965-6)
Supplement: Additional file 1: — Full list of recorded diagnoses for the outpatient surveillance sample. (DOCX 21 kb) [file 12913_2016_1965_MOESM1_ESM.docx]

**Supplementary File 1. Full list of recorded diagnoses for the outpatient surveillance sample**

| **Recorded diagnosis** | **Appears in the Standard Treatment Guidelines*** | |
| --- | --- | --- |
|  | **Yes (n)** | **No (n)** |
| Abscess | 6 |  |
| Abscess breast | 2 |  |
| Abdominal mass |  | 1 |
| Ache- back |  | 41 |
| Ache- ear |  | 2 |
| Ache- General Body (GBA) |  | 108 |
| Ache- head |  | 99 |
| Ache- lumbago |  | 2 |
| Ache- neck |  | 5 |
| Ache-stomach |  | 7 |
| Adhesions |  | 1 |
| Adhesions- hand |  | 1 |
| Amenorrhoea |  | 2 |
| Anaemia | 417 |  |
| Anaemia- in pregnancy | 8 |  |
| Anaemia- postnatal | 2 |  |
| Anaemia- severe | 25 |  |
| Anaphylaxis | 3 |  |
| Appendicitis | 2 |  |
| Arthritis | 44 |  |
| Asthma | 14 |  |
| Blood disorder |  | 1 |
| Boil | 10 |  |
| Bowel obstruction |  | 1 |
| Bronchiolitis | 18 |  |
| Bronchitis | 21 |  |
| Burns | 3 |  |
| Cellulitis | 5 |  |
| Cervicitis | 2 |  |
| Chicken pox |  | 11 |
| Chronic liver disease | 1 |  |
| Chronic lung disease | 1 |  |
| Cold | 5 |  |
| Conjunctivitis | 37 |  |
| Constipation |  | 1 |
| Cough | 480 |  |
| Cough- productive | 45 |  |
| Cough- simple | 265 |  |
| Dehydration |  | 2 |
| Dental- toothache |  | 6 |
| Dental caries |  | 6 |
| Dental- dry socket |  | 1 |
| Dental- gingivitis |  | 3 |
| Dental- oral thrush |  | 3 |
| Dental- sore gums |  | 3 |
| Dental- sore mouth |  | 1 |
| Dental- teething |  | 1 |
| Diabetes | 8 |  |
| Diarrhoea | 221 |  |
| Diarrhoea dehydrated | 15 |  |
| Diarrhoea- dysentery | 23 |  |
| Diarrhoea not dehydrated | 15 |  |
| Disability |  | 1 |
| Dizziness |  | 2 |
| Dysfunctional uterine bleeding |  | 1 |
| Ear discharge | 1 |  |
| Ear inflammation | 5 |  |
| Eczema |  | 1 |
| Engorged breast |  | 1 |
| Epilepsy |  | 3 |
| Epistaxis |  | 3 |
| Febrile convulsions/ fits |  | 3 |
| Fever | 66 |  |
| Filariasis | 1 |  |
| Flu | 233 |  |
| Food poisoning | 48 |  |
| Fracture- tibia |  | 1 |
| Gastritis | 14 |  |
| Gastroenteritis | 79 |  |
| Gland swollen |  | 2 |
| Gout | 1 |  |
| Haemoptysis |  | 1 |
| Haemorrhage |  | 1 |
| Heart burn |  | 3 |
| Heart failure | 1 |  |
| Hepatitis | 1 |  |
| Hernia |  | 1 |
| High sugar level |  | 1 |
| Hyperemesis gravidarum |  | 6 |
| Hypertension | 13 |  |
| Hyperthermia |  | 1 |
| Hypoglycaemia |  | 3 |
| Impetigo | 4 |  |
| Incomplete abortion |  | 2 |
| Infection bacterial |  | 26 |
| Infection bowel |  | 5 |
| Infection chest |  | 127 |
| Infection ear | 43 |  |
| Infection eye | 2 |  |
| Infection- neonatal |  | 5 |
| Infection- puerperal |  | 1 |
| Infection skin |  | 16 |
| Infection viral |  | 11 |
| Injury |  | 3 |
| Injury- back |  | 1 |
| Injury- eye |  | 6 |
| Injury- head |  | 2 |
| Injury- muscle strain |  | 20 |
| Injury- neck |  | 1 |
| Injury- nerve sciatica |  | 2 |
| Injury- soft tissue |  | 16 |
| Injury- tendon damage |  | 1 |
| Injury- trauma |  | 7 |
| Jaundice | 2 |  |
| Jaundice- neonatal | 1 |  |
| Joint ache | 13 |  |
| Joint- loose |  | 2 |
| Laps syndrome |  | 2 |
| Leprosy | 2 |  |
| Leukaemia |  | 1 |
| Lump on neck |  | 1 |
| Lymphs- enlarged lymph nodes/glands | 10 |  |
| Lymphs- lymphadenitis | 13 |  |
| Malaria | 16 |  |
| Malaria- severe | 7 |  |
| Malaria- treatment failure (TFM) | 5 |  |
| Malaria- uncomplicated | 482 |  |
| Malnourished |  | 4 |
| Measles |  | 2 |
| Meningitis | 13 |  |
| Meningitis cerebral | 1 |  |
| Menopause |  | 4 |
| Mucous pallor |  | 1 |
| Mumps |  | 6 |
| Nephrotic syndrome |  | 1 |
| Oedema | 2 |  |
| Otitis externa | 25 |  |
| Otitis externa (acute) | 3 |  |
| Otitis media | 219 |  |
| Otitis media (acute) | 64 |  |
| Otitis media (chronic) | 3 |  |
| Pain- abdominal | 46 |  |
| Pain- chest | 2 |  |
| Pain- epigastric |  | 11 |
| Pain- menstruation |  | 1 |
| Pertussis |  | 3 |
| Pharyngitis | 1 |  |
| Pelvic Inflammatory Disease (PID) | 12 |  |
| Pleural effusion |  | 1 |
| Pregnant |  | 12 |
| Premature labour |  | 1 |
| Pyrexia of Unknown Origin (PUO) | 399 |  |
| Vaginal bleeding | 2 |  |
| Pyelonephritis |  | 1 |
| Pyomyositis | 1 |  |
| Respiratory- Acute Respiratory Tract Infection | 2 |  |
| Respiratory- Lower Respiratory Infection (LRTI) Pneumonia | 499 |  |
| Respiratory- LRTI- Pneumonia- mild | 251 |  |
| Respiratory- LRTI- Pneumonia- moderate | 70 |  |
| Respiratory- LRTI- Pneumonia- severe | 4 |  |
| Respiratory- Respiratory Tract Infection | 11 |  |
| Respiratory- Upper Respiratory Tract Infection | 600 |  |
| Runny nose |  | 5 |
| Sepsis- neonatal | 10 |  |
| Sepsis- postnatal | 1 |  |
| Sepsis- puerperal | 2 |  |
| Sepsis- septic abortion | 1 |  |
| Sepsis- septic gallstone | 1 |  |
| Short sightedness |  | 1 |
| Sinusitis |  | 3 |
| Skin disease | 29 |  |
| Skin pustules | 1 |  |
| Skin- rash | 3 |  |
| Skin- scabies | 1 |  |
| Sore- dog bite | 1 |  |
| Sore- infected | 65 |  |
| Sore throat | 2 |  |
| Sores | 41 |  |
| Sores- anal canal | 1 |  |
| Splenomegaly | 30 |  |
| Sexually Transmitted Infection (STI) | 5 |  |
| STI- gonorrhoea | 1 |  |
| Stomatitis |  | 2 |
| Stress |  | 87 |
| Swollen breasts |  | 1 |
| Swollen groin |  | 1 |
| Swollen limbs |  | 1 |
| Tuberculosis (TB) | 11 |  |
| TB- pulmonary | 7 |  |
| Tonsillitis | 11 |  |
| Torticollis |  | 2 |
| Typhoid | 20 |  |
| Ulcer | 3 |  |
| Ulcer- peptic | 2 |  |
| Unclear |  | 2 |
| Urethritis |  | 1 |
| Urinary tract infection (UTI) | 50 |  |
| Vaginitis |  | 1 |
| Vomiting |  | 4 |
| Worms | 88 |  |
| Yaws | 13 |  |
| **Total** | **5391** | **761** |

*the number of times the diagnoses appears, whether as a first diagnosis, a second diagnosis or a third diagnosis.
